# Correlation between the level of the external wound and the internal injury in penetrating neck injury does not favour an initial zonal management approach

**DOI:** 10.1002/bjs5.50282

**Published:** 2020-06-11

**Authors:** A. S. Madsen, J. L. Bruce, G. V. Oosthuizen, W. Bekker, M. Smith, V. Manchev, G. L. Laing, D. L. Clarke

**Affiliations:** ^1^ Pietermaritzburg Metropolitan Trauma Service, Department of Surgery University of KwaZulu‐Natal, Nelson R. Mandela School of Medicine, Pietermaritzburg KwaZulu‐Natal South Africa; ^2^ Department of Surgery University of the Witwatersrand Johannesburg South Africa

## Abstract

**Background:**

Many current protocols for managing penetrating neck injuries (PNIs) still suggest zonal approaches. This study was undertaken to determine the correlation between the zone of the external wound and the level of the internal injury, and to verify whether a ‘no‐zone’ approach to PNI is valid.

**Methods:**

Patients admitted with a PNI to a tertiary trauma care centre between January 2011 and May 2018 were identified from a trauma database. Those with confirmed injury to the vascular system or an aerodigestive tract injury (ADTI) were included in the study. The medical records of each patient were reviewed with regard to the zone of the external wound and the level of internal injury, and the findings were compared.

**Results:**

In the period under review, 1075 patients were treated for a PNI. Of these, 298 (27·7 per cent) had a confirmed vascular injury or ADTI and were included in the cohort. In 176 patients (59·1 per cent) the site of the internal injury was in the same zone as the external wound. In a further 70 patients (23·5 per cent) there was no correlation between the site of the internal injury and the external wound, and in the remaining 52 patients (17·4 per cent) the correlation could not be determined. In this cohort, all clinically assessable patients with significant injuries had either physical signs suggestive of injury or deep surgical emphysema on radiological examination.

**Conclusion:**

An approach to PNI based on zones is questionable, and this study supports a no‐zone approach based on imaging guided by clinical examination.

## Graphical abstract

This large single‐centre review of patients treated for penetrating neck injuries over a 7·5‐year period examined whether the traditional zonal approach to penetrating neck injury still holds relevance in the contemporary era or whether a ‘no‐zone’ approach is more valid. The correlation between the zone of the external wound and the level of the internal injury in patients with confirmed vascular or aerodigestive tract injuries was determined. It was concluded that the traditional zonal approach to penetrating neck injury is questionable in the current era.

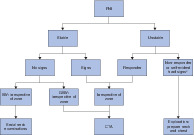

Rejecting the zonal approach

## Abstract (es)

**Antecedentes:**

Muchos protocolos actuales para el manejo de lesiones penetrantes en el cuello (*penetrating neck injury,* PNI) aún proponen un enfoque zonal. Este estudio se llevó a cabo para determinar la correlación entre la zona de la herida externa y el nivel de la lesión interna y para comprobar si sería válido un enfoque "sin zonas" para la PNI.

**Métodos:**

Los pacientes con PNI ingresados en un centro terciario de traumatología entre enero de 2011 y mayo de 2018 fueron identificados a partir de la base de datos del centro. Se incluyeron pacientes con lesión confirmada vascular o lesión del tracto aero‐digestivo (*aero‐digestive tract injury*, ADTI). Se revisaron las historias clínicas de cada paciente con respecto a la zona de la herida externa y el nivel de lesión interna, comparándose dichos hallazgos.

**Resultados:**

En el período de estudio, 1.075 pacientes fueron tratados por una PNI. De estos, 298 (27,7%) tenían una lesión vascular o una ADTI confirmadas y se incluyeron en la cohorte. En 176 pacientes (59,1%), la lesión interna estaba localizada en la misma zona de la herida externa. En otros 70 pacientes (23,5%), no hubo correlación entre la localización de la lesión interna y la herida externa y en los 52 pacientes restantes (17,4%) no se pudo determinar dicha correlación. En esta cohorte, todos los pacientes clínicamente evaluables con lesiones significativas presentaban signos físicos sugestivos de lesión o enfisema profundo con indicación quirúrgica en el examen radiológico.

**Conclusión:**

El enfoque de la PNI basado en zonas es cuestionable y este estudio apoya un enfoque de "sin zonas" basado en pruebas de imágen basadas en los hallazgos clíncos.

## Introduction

The management of penetrating neck injury (PNI) has undergone a steady evolution over the past 60 years. Mandatory exploration of all wounds penetrating the platysma muscle has been superseded by a selective non‐operative management approach based on clinical examination and imaging^1^, ^2^, ^3^, ^4^, ^5^, ^6^, ^7^, ^8^, ^9^, ^10^, ^11^, ^12^, ^13^, ^14^, with one of the original drivers of this approach being the concept of the zones of the neck. This was first proposed by Monson and colleagues^3^ in 1969 and later modified by Roon and Christensen^4^ in 1979. This concept emerged with the development of better imaging modalities and from the observation that mandatory exploration resulted in an unacceptably high negative exploration rate, which, especially for the lower and upper zones of the neck, was associated with a not insignificant morbidity rate^2^, ^3^, ^4^, ^10^.

The zonal approach was used to decide on the need for imaging and to guide the surgeon on the operative exposure required if exploration was undertaken. Operative exploration without imaging was suggested only for wounds to zone II of the neck, as the structures in zone II are easily accessible, whereas wounds to zone I and III were imaged before operative exploration^3^, ^4^.

Since this approach was first popularized 40 years ago there have been dramatic advances in imaging and in endovascular therapeutic techniques. Modern contrast CT angiography (CTA) is non‐invasive, extremely reliable and easy to perform, and has all but eliminated the need for invasive formal catheter‐directed angiography^15^, ^16^, ^17^, ^18^. Furthermore, the correlation between the zone of the external wound and the level of the internal injury has been questioned recently^19^. This has led some authors to suggest a so‐called ‘no‐zone’ approach based solely on the clinical signs and findings on CTA^19^, ^20^, ^21^, ^22^.

This study aimed to review whether the traditional zonal approach to PNI still holds relevance in the contemporary era or whether a no‐zone approach is more valid, by determining the correlation between the zone of the external wound and the level of the internal injury in PNI.

## Methods

The study was undertaken at the Pietermaritzburg Metropolitan Trauma Service (PMTS) in Pietermaritzburg, KwaZulu‐Natal, South Africa. The PMTS provides trauma care for the city of Pietermaritzburg, with one million inhabitants, and tertiary trauma care for the entire rural western third of the Province of KwaZulu‐Natal, with a further two million inhabitants. The PMTS maintains a unique hybrid electronic medical registry (HEMR), which captures demographic and clinical data in real time on all admitted injured patients^23^. The HEMR is managed and quality‐controlled by designated attending trauma surgeons. Ethical approval was granted for the registry and use of its data for clinical research by the Biomedical Research Ethics Committee of the University of KwaZulu‐Natal (reference number BE 207/09 and BCA 221/13).

All patients admitted with a PNI from January 2011 to May 2018 were identified from the HEMR. Those with a confirmed injury to the vascular system or an aerodigestive tract injury (ADTI) were included in the study. Each patient's medical record was reviewed to determine the zone of the external wound and the level of the internal injury, in order to assess correlation.

### Management algorithms at the trauma service

All patients with a PNI are managed at the PMTS according to Advanced Trauma Life Support® (ATLS®; American College of Surgeons) principles^24^. Haemodynamically unstable patients who do not respond to resuscitation, which might include an initial attempt at arresting exsanguinating bleeding with a Foley catheter (so‐called Foley catheter balloon tamponade)^25^, are expedited to the operating room. Stabilized patients undergo detailed clinical examination. All stable patients, with no ongoing bleeding or expanding haematomas, but with either a soft or stable hard sign of vascular injury or a sign of ADTI, are investigated further with CTA and/or water‐soluble contrast swallow irrespective of the zone involved. Patients with an impaired level of consciousness and all stable patients with gunshot wounds (GSW) to the neck irrespective of zone also undergo imaging. Patients with stab wounds (SW) who are alert and with no signs of injury are admitted for observation with serial neck examination and are not imaged routinely.

### Zones of the neck

**Figure 1:**
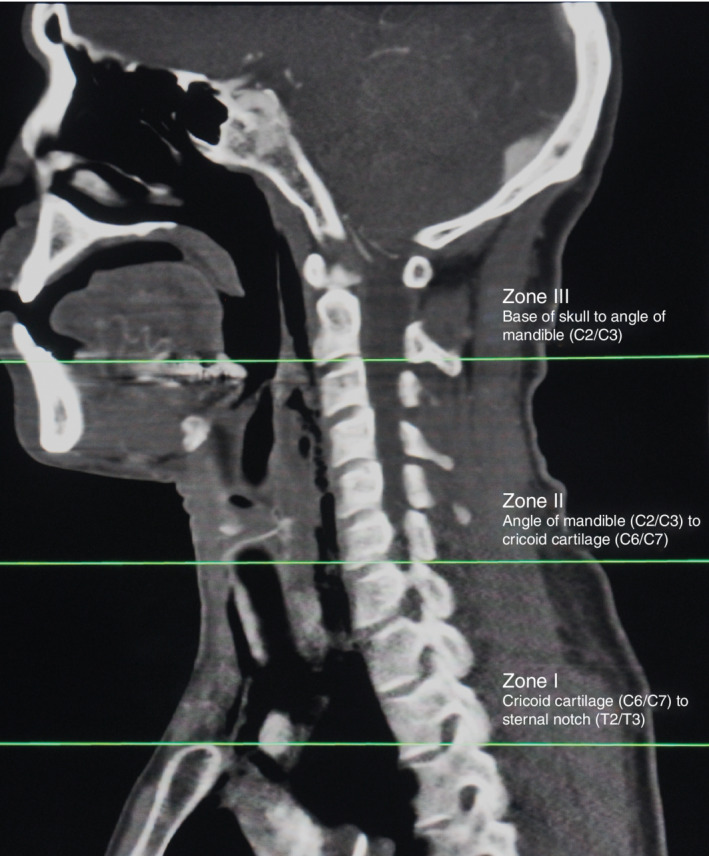
Roon and Christensen's classification^4^ of neck zones as seen on sagittal cervical CT angiography

Roon and Christensen's classification of the neck zones^4^ (*Fig*. 1) was used, and the vascular and aerodigestive contents of these zones were considered to be: zone I, extending from the sternal notch to the cricoid cartilage (T3/T2–C6/C7) and containing the cervical part of the oesophagus and trachea, proximal common carotid artery (CCA) but excluding the most proximal part of the left CCA, proximal vertebral artery (VA), distal jugular vein, subclavian artery (SCA) but excluding the first part of the left SCA, subclavian vein (SCV) and proximal axillary artery and vein; zone II, extending from the cricoid cartilage to the angle of the mandible (C6/C7–C3/C2) and containing the lower part of the oropharynx, hypopharynx, larynx, distal CCA, proximal internal carotid artery (ICA), proximal external carotid artery (ECA), middle part of the jugular vein and middle part of the VA; zone III, extending from the angle of the mandible to the base of the skull (C3/C2 to base of skull) and containing the upper part of the oropharynx, nasopharynx, and distal part of the ICA, ECA and VA, proximal part of the jugular vein, facial vessels and maxillary vessels.

The zones of the external wounds were retrieved from the admission records in the HEMR and outpatient notes. The levels of internal injuries were determined by reviewing results of all imaging, such as CTA, water‐soluble contrast swallow, endoscopy, formal angiography and operative records. When results from both imaging and exploration were available, operative records were considered the standard.

When the level of the internal injury was in the same zone as the external wound, the patient was classified as ‘correlating’. Patients with internal injuries found to be outside the zone of the external wound were classified as ‘not correlating’ and further subdivided as to whether the injury was above or below the zone of the external wound. Patients in whom the level of the internal injury or the zone of the external wound could not be fully determined were classified as ‘indeterminate’.

Patients with multiple external zonal involvements were classified as correlating only if the level of the internal injury was within the trajectory caused by a single transcervical penetration, or if the external wound was a single large wound extending over more than one zone and the level of the internal injury was found to be within one of these zones. Likewise, patients with multiple zonal involvements were classified as not correlating if the level of an internal injury was found to be outside any of the involved external zones. Patients with multiple external unpaired wounds, preventing any meaningful assessment of correlation, were classified as indeterminate.

In addition, data pertaining to the mechanism of injury, Injury Severity Score (ISS), clinical signs of injury, investigations, management and outcome were analysed for the cohort.

### Statistical analysis

The χ^2^ test and the Fisher's exact test were used, where appropriate, to examine relationships between categorical variables. The Wilcoxon test was used for continuous variables. The significance level was *P* < 0·050.

Logistic regression models and odds ratios (ORs) with 95 per cent c.i. were calculated for significant relationships using R version 3.5.2 (The R Foundation for Statistical Computing, Vienna, Austria), with a significance level of *P* < 0·050.

## Results

During the study period, 1075 patients were treated for a PNI, of whom 298 (27·7 per cent) had a confirmed vascular injury or ADTI and were thus included. Some 270 (90·6 per cent) of the patients were men and the median age was 27 (i.q.r. 23–33·8) years. The mechanism of injury was SW in 241 patients (80·9 per cent) and a GSW in 57 (19·1 per cent). The median ISS was 16 (i.q.r. 9–20).

The distribution of the neck zones of the external wounds was: zone I in 106 patients (35·6 per cent), zone II in 114 (38·3 per cent) and zone III in 39 (13·1 per cent). The remaining 39 patients (13·1 per cent) had involvement of multiple zones, of which 17 were paired transcervical wounds (16 GSW), 15 were multiple unpaired wounds, and seven were single wounds extending over multiple zones.

The corresponding zonal levels of the internal injuries were: zone I in 120 patients (40·3 per cent), zone II in 86 (28·9 per cent) and zone III in 26 (8·7 per cent). Multiple zones were involved in 12 patients (4·0 per cent) and the mediastinum in 15 (5·0 per cent). The level of the internal injury was indeterminate in 39 patients (13·1 per cent). For 30 of these patients, this was due to vascular injuries identified on CTA as thrombosed vessels, and which extended over more than one zone (internal jugular vein (IJV), 24; VA, 5; ICA/IJV, 1); these were managed without surgery. For one patient, a CTA‐identified pharyngeal injury secondary to a GSW was due to the lack of further assessment of the level with a contrast swallow. A further five patients did not have the exact level of the internal injury described at the time of surgery (IJV, 2; CCA/IJV, 1; anterior jugular vein, 1; external jugular vein, 1), and three patients exsanguinated from vascular injury before reaching the operating room.

Some 198 patients sustained 256 vascular injuries, 93 patients had 94 digestive tract injuries, and 52 patients had 54 airway injuries secondary to the PNI (*Table* 1). In total, 136 patients (43·6 per cent) had additional extracervical penetrating trauma (*Table* 2).

**Table 1:** Vascular and aerodigestive tract injuries secondary to penetrating neck injury in 298 patients

Vascular (*n* = 198)		Digestive (*n* = 93)		Airway (*n* = 52)	
**Venous**	*n* = 106	Oesophagus	29 (31)	Trachea	33 (63)
Internal jugular vein	72 (36·4)	Hypopharynx	48 (52)	Larynx	21 (40)
Subclavian vein	20 (10·1)	Oropharynx	17 (18)		
External/anterior jugular vein	10 (5·1)				
Innominate vein	3 (1·5)				
Superior vena cava	1 (0·5)				
**Arterial**	*n* = 150				
Arch of aorta	1 (0·5)				
Descending aorta	1 (0·5)				
Pulmonary artery and branch	3 (1·5)				
Innominate artery	8 (4·0)				
Subclavian artery	36 (18·2)				
Proximal axillary artery	6 (3·0)				
Common carotid artery	30 (15·2)				
Internal carotid artery	9 (4·5)				
External carotid artery	7 (3·5)				
Vertebral artery	23 (11·6)				
Intrathoracic artery	2 (1·0)				
Thyrocervical trunk	2 (1·0)				
Costocervical artery	2 (1·0)				
Dorsal scapular artery	2 (1·0)				
Suprascapular artery	1 (0·5)				
Deep cervical artery	1 (0·5)				
Inferior thyroid artery	1 (0·5)				
Maxillary artery	5 (2·5)				
Facial artery	5 (2·5)				
Lingual artery	2 (1·0)				
Unconfirmed vessels*	3 (1·5)				

Values in parentheses are percentages.

* These patients exsanguinated before exploration and specific vessels could not be confirmed.

**Table 2:** Concomitant cervical and extracervical injuries in 298 patients

**Cervical (*n* = 298)**		**Maxillofacial (*n* = 65)**		**Head (*n* = 28)**		**Chest (*n* = 124)**		**Abdomen (*n* = 16)**		**Extremities (*n* = 36)**	
Wound	298 (100)	Wound	60 (92)	Wound	20 (71)	Wound	57 (46)	Wound	16 (100)	Wound	36 (100)
Cervical spinal fracture	31 (10·4)	Facial fracture	38 (58)	Skull fracture	5 (18)	Haemopneumothorax	84 (68)	Stomach	1 (6)	Brachial artery	1 (3)
Cervical spinal cord	10 (3·4)	Facial nerve	6 (9)	Intracranial bleed or contusion	5 (18)	Haemopneumomediastinum	40 (32)	Duodenum	2 (13)	Radial nerve	1 (3)
Brachial plexus	13 (4·4)	Eye	3 (5)	Infarction	8 (29)	Pneumopericardium	2 (2)	Small bowel	1 (6)	Fracture	6 (17)
Thyroid	4 (1·3)					Cardiac	2 (2)	Liver	2 (13)		
RLN	2 (0·7)					Rib fracture	3 (2)	Pancreas	1 (6)		
Phrenic nerve	1 (0·3)					Clavicle fracture	3 (2)	Hepatic artery	1 (6)		
Vagus nerve	1 (0·3)					Scapula fracture	3 (2)	Portal vein	1 (6)		
						Thoracic spinal fracture	3 (2)	Lumbar spinal cord	1 (6)		

Values in parentheses are percentages. RLN, recurrent laryngeal nerve.

### Clinical signs of injury

Of the 198 patients with vascular injury, 65 (32·8 per cent) had hard signs, 109 (55·1 per cent) had soft signs, 22 (11·1 per cent) had no clinical signs, and there were no assessable records regarding clinical signs for the other two patients (1·0 per cent).

Twelve of the 22 patients with no clinical signs of injury had venous injuries to the IJV or SCV diagnosed on CTA. Eleven of these were managed conservatively; only one with a venous injury, which was diagnosed incidentally during exploration for tracheal injury, was managed by surgery. Ten of the 22 patients with no clinical signs had arterial injuries (six from GSW and four from SW), but only two were isolated arterial injuries that could be assessed fully (dorsal scapular artery and lingual artery). These two injuries were managed conservatively. The remaining eight patients had concomitant airway injuries requiring urgent intubation, a reduced Glasgow Coma Scale (GCS) score, neurogenic shock, or an abdominal vascular injury. These associated injuries made it difficult to assess thoroughly for clinical signs of a vascular injury. Two of these eight patients were managed by surgery (1 ICA and 1 innominate artery), two by endovascular intervention (1 VA and 1 VA/CCA), and four conservatively (3 VA and 1 SCA).

Of the 93 patients with digestive tract injury, 77 (83 per cent) had signs of injury and the other 16 (17 per cent) had no recorded clinical signs. Three of the patients with no signs were rushed to the operating room for concomitant injuries (stabbed heart and exsanguinating vascular injuries), and a further seven had a reduced GCS score, eliminating proper clinical examination. Only six patients (6·5 per cent) without clinical signs of digestive tract injury were fully assessable (2 oropharynx, 1 hypopharynx and 3 oesophagus), and all had deep surgical emphysema on CTA or cervical X‐ray. Only two of these were managed by surgery (1 hypopharynx and 1 oesophagus with concomitant CCA and IJV injury).

Of the 52 patients with airway injury, 50 (96 per cent) had clinical signs of injury and only two had no recorded clinical signs. These two patients had visible minor tracheal injuries on CTA and deep surgical emphysema; both were managed conservatively.

### Investigations and management

On admission, 229 patients were investigated with CTA, 29 with formal angiography, 145 with contrast swallow, and 37 with endoscopy (20 upper gastrointestinal endoscopy, 13 bronchoscopy and 4 laryngoscopy). A total of 178 patients (59·7 per cent) were managed by surgery (156 patients) or endovascular intervention (22), and the remaining 120 (40·3 per cent) without surgery. Fifty‐six patients (18·8 per cent) had emergency surgery with no prior imaging (*Table* 3). For 17 of these patients the indication was a threatened airway, and for 35 it was haemodynamic instability and/or uncontrollable bleeding from the neck wound (isolated arterial injury in 10, isolated venous injury in 15, mixed arterial and venous injury in 10). A further two patients were expedited to the operating room owing to concomitant cardiac injuries, during which the neck was also explored, and two patients were taken directly to the operating room because of clinically obvious pharyngeal injury.

**Table 3:** Comparison of management and indications for emergency surgery in patients with correlating and non‐correlating injuries

	*n*	Correlating (*n* = 176)	Non‐correlating (*n* = 70)	*P* ‡	Odds ratio*, §	Indeterminate† (*n* = 52)
**Management**						
Surgery or endovascular	178	112 (62·9)	52 (29·2)	0·110		14 (7·9)
Emergency surgery, no imaging	56	30 (54)	20 (36)	0·043	0·51 (0·27, 1·00)	6 (11)
**Indication for emergency surgery**						
Airway compromise	17	9 (53)	8 (47)	0·465		0 (0)
Bleeding from artery	10	6 (60)	4 (40)	0·100		0 (0)
Bleeding from vein	15	9 (60)	2 (13)	0·163		4 (27)
Bleeding from artery and vein	10	4 (40)	4 (40)	0·697		2 (20)
Cardiac injury	2	0 (0)	2 (100)	0·155		0 (0)
Pharyngeal injury	2	2 (100)	0 (0)	0·510		0 (0)

Values in parentheses are percentages unless indicated otherwise;

* values in parentheses are 95 per cent confidence intervals.

† Results for indeterminate group are shown but were not compared with the other two groups.

‡ χ^2^ or Fisher's exact test.

§ Calculated for significant relationships (where *P* < 0·050).

### Outcome

The median length of stay was 6 (i.q.r. 3–12) days, and 105 patients (35·2 per cent) required intensive/high care. The mortality rate was 7·0 per cent (21 patients) and there were no unavoidable deaths. Sixty‐four (23·1 per cent) of the 277 surviving patients had the following morbidities: 15 pharyngeal/oesophageal leaks, eight retained haemothoraces, eight pneumonia, seven cerebral infarcts, five mediastinitis/retropharyngeal abscesses, four episodes of rebleeding (3 postoperative, 1 non‐operative), four graft occlusions, two cases of line sepsis, two breakdowns of small bowel/duodenal repair, two cases of osteomyelitis (sternum, mandible), two pericardial effusions, one bullet embolectomy (pulmonary artery), one occlusion of a temporary intravascular shunt, one stent occlusion, one cutaneous parotid fistula, and one bronchopleural fistula.

### Correlation between zone of the external wound and the internal injury

In 176 patients (59·1 per cent) the level of the internal injury correlated with the zone of the external wound. In a further 70 patients (23·5 per cent), there was no correlation between the level of the internal injury and the external wound, with 62 of these having the internal injury below the zone of the external wound, seven above, and one both above and below.

For 52 patients (17·4 per cent) it was not possible to determine the correlation; these were classified as indeterminate. The main reasons for this were conservative management of thrombosed, injured vessels seen on CTA in 27 patients, and multiple external zonal involvement precluding an accurate determination of correlation in 15. In five patients, the zonal level of the internal injuries was not described at the time of surgery. Three patients died from exsanguination before reaching the operating room, and the correlation could not be determined with certainty in the final two patients owing to adjacent concomitant penetrating trauma to the upper chest.

Regarding the zonal location of the external wounds, the only statistically significant differences observed between the two groups (correlating and non‐correlating injuries) were in patients with zone II wounds (*P* = 0·032) and those with multiple zonal involvement (*P* = 0·015) (*Table* 4). Patients with zone II wounds were approximately half as likely to have correlating injuries as those with wounds in other zones (OR 0·54, 95 per cent c.i. 0·31 to 0·95), and patients with multiple external zonal involvement were 8·5 times more likely to have correlating injuries than those with wounds in other zones (OR 8·40, 1·70 to 151·00).

**Table 4:** Comparison of different variables in patients with correlating and non‐correlating injuries

	*n*	Correlating (*n* = 176)	Non‐correlating (*n* = 70)	*P* ¶	Odds ratio† **	Indeterminate‡ (*n* = 52)
**External zone**						
I	106	74 (69·8)	24 (22·6)	0·262		8 (7·5)
II	114	62 (54·4)	35 (30·7)	0·032	0·54 (0·31, 0·95)	17 (14·9)
III	39	21 (54)	10 (26)	0·616		8 (21)
Multiple	39	19 (10·8)	1 (1)	0·015	8·40 (1·70, 151·00)	19 (37)
**Mechanism**						
Stab wound	241	139 (57·7)	58 (24·1)	0·492		44 (18·3)
Gunshot wound	57	37 (65)	12 (21)			8 (14)
**Injuries sustained**						
Vascular	198	*n* = 110	*n* = 40	0·437		*n* = 48§
Arterial	92	57 (62)	22 (24)	0·885		13 (14)
Venous	65	28 (43)	8 (12)	0·370		29 (45)
Arterial and venous	38	25 (66)	10 (26)	0·987		3 (8)
Digestive	93	63 (68)	27 (29)	0·683		3 (3)
Airway	52	30 (58)	19 (37)	0·074		3 (6)
**Signs of vascular injury**						
Vascular signs	174	*n* = 97	*n* = 35	0·910		*n* = 42
Hard	65	32 (49)	17 (26)	0·102		16 (25)
Soft	109	65 (59·6)	18 (16·5)			26 (23·9)
**SBP (mmHg)**						
< 100	45	23 (51)	13 (29)	0·271		9 (20)
≥ 100	253	153 (60·5)	57 (22·5)			43 (17·0)
**ISS** *	–	15 (9–18)	17·5 (11–25)	0·003#	0·96 (0·93, 0·98)	11 (9–20)

Values in parentheses are percentages unless indicated otherwise;

* values are median (i.q.r.);

† values in parentheses are 95 per cent confidence intervals.

‡ Results for indeterminate group are shown but were not compared with the other two groups.

§ Includes three patients who died from exsanguination before exploration could be performed. SBP, systolic blood pressure; ISS, Injury Severity Score.

¶ χ^2^ or Fisher's exact test, except

#Wilcoxon test.

** Calculated for significant relationships (where *P* < 0·050).

Regarding the mechanism of injury, patients with GSW were more likely to have correlating injuries than those with SW, but the difference was not statistically significant (*P* = 0·492). Similarly, no significant differences were observed between the two groups of patients with regard to vascular injury or ADTI, and whether hard or soft signs of vascular injury were present. Patients presenting in haemodynamic shock (defined as a systolic BP below 100 mmHg) were less likely to have correlating injuries, but this difference was not statistically significant (*P* = 0·271).

Although the median ISS was lower in patients with correlating injuries (15 (i.q.r. 9–18) than in those with non‐correlating injuries (17·5 (11–25)) and the difference was statistically significant (*P* = 0·003), the OR was close to 1·00 (OR 0·96, 95 per cent c.i. 0·93 to 0·98).

Patients who went straight to emergency surgery without prior imaging were less likely to have correlating than non‐correlating injuries. This difference was just statistically significant (*P* = 0·043, χ^2^ test), but, despite an OR of 0·51, the upper limit of the 95 per cent c.i. was 1·00 (*Table* 3).

## Discussion

The correlation between the zone of the external wound and the internal injury was only 59·1 per cent in this study. Patients with external wounds to zone II were approximately half as likely to have correlating injuries as those with external wounds to other zones. This finding could be due to the fact that correlation was determined from the presence of vascular and ADTIs only. Patients with external wounds to zone II could have these injuries in the same zone as well as in the zone above and below, whereas patients with zone III external wounds alone could have these injuries in the same zone or below. Although patients with external wounds in zone I could also have ADTI or vascular injuries below this zone (the mediastinum), the bony inferior border of this zone, composed of the clavicles and sternum, possibly prevented some of these injuries, thereby accounting for the observed lower rate of correlating injuries in zone II.

In nearly one‐quarter of the cohort there was no correlation between the level of the external wound and the internal injury, with nearly all of these patients having the internal injury below the zone of the external wound. This is probably accounted for by a downwards‐stabbing trajectory, and the majority of PNI was caused by SW rather than GSW. Interestingly, patients with GSW did not have a lower rate of correlating injury than those with SW (64·9 *versus* 57·7 per cent respectively). A possible contributing factor to this finding could be that a large proportion of patients with multiple external zonal involvement had paired transcervical wounds sustained from single gunshots to the neck, and hence the trajectory of many GSW could be determined and an assessment made for correlation. The presence of clinical signs of vascular injury and the injury severity judged by the ISS did not significantly affect the proportions of patients with correlating injuries.

The zonal approach to PNI was developed as a clinical decision guide for haemodynamically stable patients. It was considered diagnostically beneficial indiscriminately to explore all wounds in the easily accessible zone II and to image injuries to zones I and III with invasive imaging modalities. This approach was, however, not without associated risks and morbidities. The negative exploration rate was undeniably high, the risk of missing an injury persisted despite neck exploration, and the risk of a complication arising from the use of various invasive imaging techniques was not negligible^3^, ^4^. Although this approach to PNI was appropriate for its era, it was expensive, invasive, labour‐intensive and, for the most part, of low clinical yield.

Since then, data have accumulated supporting an approach to patients with PNI based increasingly on the clinical signs of injury (so‐called hard, soft or no signs) and simple radiological imaging with X‐rays^6^, ^7^, ^8^, ^9^, ^10^, ^11^, ^12^, ^13^, ^14^, ^16^. This has been more widely accepted for zone II injuries, although some literature^6^, ^7^, ^8^, ^10^, ^11^, ^12^ suggests this is also a reasonable approach for zones I and III. Although alterations have been made to the zonal approach, and indiscriminate exploration of all zone II wounds is now considered obsolete, zonal approaches of various kinds are still being suggested in recent trauma protocols for PNI^17^, ^26^, ^27^. Despite this, there is only one article^19^ in the literature that specifically investigated the correlation between the zone of the external wound and the level of the internal injury. In this study, which included only 38 patients with confirmed injuries, the authors concluded that the correlation was poor (79 per cent) and suggested a no‐zone approach. The results of the present study strongly support this conclusion.

The most recent and significant development in the management of patients with PNI has been the adoption of CTA for assessment. CTA has excellent sensitivity and specificity for vascular injuries, as well as a high negative predictive value for ADTI^16^, ^17^, ^18^, ^28^. Although CTA oesophagography has been described for the detection of both vascular injury and ADTI in PNI, there is little experience with this^29^. A CT‐identified bullet trajectory without proximity to vital structures has been suggested as a valid radiological finding to exclude ADTI^30^. However, it is difficult to delineate the trajectory of a SW, and a previous publication^28^ demonstrated that the absence of a radiological finding of deep surgical emphysema virtually excludes any significant ADTI. Patients with clinical signs of ADTI or deep surgical emphysema need further investigation with a water‐soluble contrast swallow. This is generally sufficient to rule out any significant digestive tract injury, and reserves endoscopy for the comatose or uncooperative patient with suspected ADTI^31^.

It is controversial whether all stable patients with PNI should be screened with CTA. CTA is non‐invasive and therefore avoids many of the complications associated with invasive imaging modalities. However, CTA does make use of potentially nephrotoxic and cytotoxic contrast, and furthermore it results in a sizeable number of false‐positive arterial studies (13·3–14·8 per cent)^16^, ^18^ and has a low positive predictive value for ADTI (30 per cent)^28^. The literature seems to suggest that it is probably superfluous to image asymptomatic patients irrespective of zones of injury, at least in busy trauma centres, although the opposite might be true in surgical departments with no experience of assessing patients with PNI^16^, ^18^. The results of the present study suggest that significant vascular or ADTIs in patients who are alert and who have no distracting injuries can be identified by physical examination combined with radiological imaging for the exclusion of deep surgical emphysema. If the patient is symptomatic but stable, further imaging is mandated.

**Figure 2:**
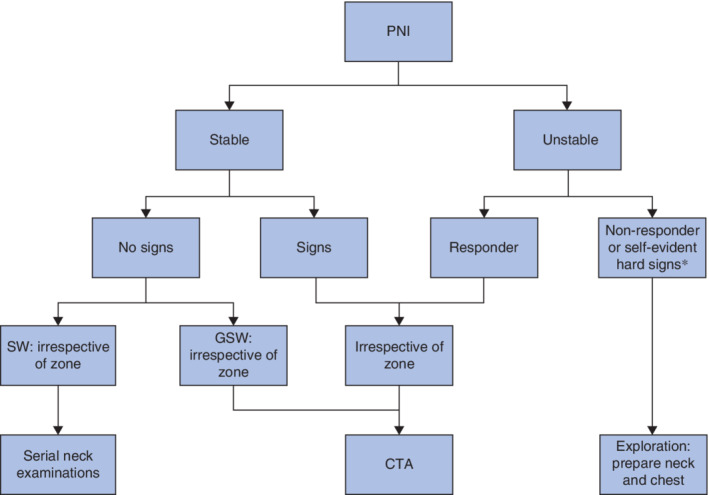
Suggested no‐zone approach to penetrating neck injury *Exsanguinating haemorrhage not amenable to Foley catheter balloon tamponade or rapidly expanding neck haematoma. PNI, penetrating neck injury; SW, stab wound; GSW, gunshot wound; CTA, CT angiography.

The haemodynamic status of the patient remains the most important determinant in the management of patients with PNI. Non‐responders to resuscitative measures need urgent exploration. Obtaining proximal and distal vascular control might very well require surgical access outside the corresponding zone of the external wound of the neck, and prepping and draping of the patient should always allow access to the chest. This study supports an initial no‐zone approach to PNI with imaging and further management guided by the presence of clinical findings of injury, irrespective of the zone involved in the external injury (*Fig*. 2). However, with today's widespread use of CTA for PNI, the increasing role of endovascular intervention, and the continuous drive for progressively more conservative management protocols, the internal injury should be described according to its zonal level, as this is one of the primary denominators guiding the choice of intervention in the haemodynamically stable patient.

## Disclosure

The authors declare no conflict of interest.
